# Object-based selection in visual working memory

**DOI:** 10.3758/s13423-021-01971-4

**Published:** 2021-07-13

**Authors:** Yin-ting Lin, Garry Kong, Daryl Fougnie

**Affiliations:** 1 Department of Psychology, New York University Abu Dhabi, Saadiyat Island, Abu Dhabi, United Arab Emirates; 2 Waseda Institute for Advanced Study, Waseda University, Tokyo, Japan

**Keywords:** Visual working memory, Object-based attention

## Abstract

Attentional mechanisms in perception can operate over locations, features, or objects. However, people direct attention not only towards information in the external world, but also to information maintained in working memory. To what extent do perception and memory draw on similar selection properties? Here we examined whether principles of object-based attention can also hold true in visual working memory. Experiment 1 examined whether object structure guides selection independently of spatial distance. In a memory updating task, participants encoded two rectangular bars with colored ends before updating two colors during maintenance. Memory updates were faster for two equidistant colors on the same object than on different objects. Experiment 2 examined whether selection of a single object feature spreads to other features within the same object. Participants memorized two sequentially presented Gabors, and a retro-cue indicated which object and feature dimension (color or orientation) would be most relevant to the memory test. We found stronger effects of object selection than feature selection: accuracy was higher for the uncued feature in the same object than the cued feature in the other object. Together these findings demonstrate effects of object-based attention on visual working memory, at least when object-based representations are encouraged, and suggest shared attentional mechanisms across perception and memory.

## Introduction

Visual attention selects salient or behaviorally relevant objects, resulting in faster and more accurate responses to those objects at the expense of other information in the environment (Duncan, 1984; Egly et al., 1994; Maunsell & Treue, 2006; Posner, 1980). But what happens when information is no longer available to perception? We can temporarily hold task-relevant information in visual working memory (VWM), and recent research has suggested that selective attention mechanisms also operate in working memory (Chun et al., 2011; Kiyonaga & Egner, 2013). For example, cueing an item in VWM improves performance for that item, i.e., the retro-cue effect (Griffin & Nobre, 2003; Landman et al., 2003; Souza & Oberauer, 2016). Similarities in the effects of selection in perception and working memory are consistent with theoretical accounts suggesting a close relationship between attention and working memory (Cowan et al., 2005; Oberauer, 2019).

However, the exact nature in which attention and working memory interact is still unclear. Research has explored whether visual attention and VWM share limited resources (Cowan, 2001; Rensink, 2000; but see Fougnie & Marois, 2006), have common neural underpinnings (Awh et al., 1999; Awh & Jonides, 2001; Gazzaley & Nobre, 2012; Nobre et al., 2004), or rely on a common template (Kong et al., 2020; Olivers et al., 2011), mostly to ambiguous results. Work addressing whether selection in perception and VWM draw on similar representational properties (Kong & Fougnie, 2019) provides another avenue to investigate this question.

Extensive work has shown that multiple forms of attention exist in perception. In light of the debate on whether memory and perception share representation content (Harrison & Tong, 2009; Serences et al., 2009; Xu, 2017), it is important to examine whether this division between types of attention in perception also applies to VWM. Yet previous studies addressing this have focused on spatial selection (e.g., Bloem et al., 2018; Fang et al., 2019; Sahan et al., 2016; Souza et al., 2018). Given that early models of visual attention were predominantly focused on spatial properties (Downing & Pinker, 1985; Eriksen & Yeh, 1985; Posner et al., 1980), this is hardly surprising.

However, a considerable number of studies have since found that attention operates on non-spatial representations, such as features (Maunsell &Treue, 2006) or objects (for reviews, see Chen, 2012; Scholl, 2001). In perception, paradigms of object-based attention have shown enhanced performance for features on the same object, compared to those on overlapping or equidistant locations on different objects (Duncan, 1984; Egly et al., 1994), demonstrating a same-object advantage that is independent of space-based attention (but see Donovan et al., 2017; Vecera, 1994). Furthermore, this object-based mechanism was distinguished from feature-based selection, as attending to one feature of an object also enhances processing for other object features (Ernst et al., 2013; O’Craven et al., 1999; Schoenfeld et al., 2014).

Object-based attention is especially important here, as there are reasons to suspect that selective mechanisms in VWM can operate over objects versus features or locations. Some suggest that objects are fundamental units of memory representations (Irwin & Andrews, 1996; Luck & Vogel, 1997; Vogel et al., 2001; but see Bays et al., 2009; Fougnie & Alvarez, 2011). Accordingly, research has suggested possible object-based effects within VWM (Awh et al., 2001; Bao et al., 2007; Gao et al., 2017; Hajonides et al., 2020; Matsukura & Vecera, 2009; Peters et al., 2015; Sahan et al., 2020; Woodman & Vecera, 2011). For example, Woodman and Vecera (2011) found that participants were less accurate when switching between different objects during memory retrieval. However, these studies often overlooked the potential contribution of location to object-based effects. Recent work has shown the importance of location in feature binding in memory (Golomb et al., 2014; Kovacs & Harris, 2019; Pertzov & Husain, 2014; Schneegans & Bays, 2017) and even suggested that observed object-based benefits arise from effects of spatial selection (Wang et al., 2016). Given that others did not find object-based attentional effects in VWM (Ko & Seiffert, 2009), it is important to isolate object-based benefits from space-based effects.

Here we investigate whether object-based effects in visual attention – beyond that which can be explained by spatial or featural attention – also apply when information is selected and updated in VWM. Experiment 1 used a memory updating task, in which participants updated equidistant features in same or different objects, to examine whether memory selection is also faster for features on the same object. Experiment 2 examined whether selection of a feature automatically leads to selection of other features within the same object by manipulating the relevance of both the object and the feature dimension (color or orientation) in a retro-cue task. Importantly, we presented objects at overlapping locations to control for location-based effects. If object-based representations guide memory selection in a similar way, selecting a feature should also facilitate selection of another feature in the same object, regardless of whether they share the same location or feature dimension.

## Experiment 1

Egly et al. (1994) demonstrated object-based attentional effects in a spatial cueing paradigm (for reviews, see Reppa et al., 2012; Shomstein, 2012). Cueing one end of one rectangular bar facilitates detection of invalid targets at the opposite end of the cue, compared to those on a different rectangle. Because invalid targets in both the same and different rectangles were equidistant from the cue, their findings demonstrate a same-object benefit that cannot be attributed to effects of spatial proximity.

Here we used a memory updating task to examine whether a similar selection benefit occurs in VWM. Participants memorized two rectangle bars with colored ends. Subsequently, participants updated colors of two rectangle ends that could be on the same bar, at equidistant locations on different bars, or diagonally located on different bars. As in previous memory updating studies (Kong & Fougnie, 2019), we measured reaction times in a self-paced updating procedure to assess which items are selected more efficiently. Finally, participants were tested on their updated memory in a change-detection display, in which we scrambled the location and diminished the size of bars to encourage encoding of objects (rather than spatial positions).

### Method

#### Participants

Eighteen students (15 female, 3 male; mean age 19.72 years, age range: 18–23 years) at New York University Abu Dhabi participated in Experiment 1 in exchange for course credit or allowance of 50 AED per hour. One participant had an accuracy rate of 50% and was replaced.

To determine the sample size required for paired *t*-tests, we conducted power analysis using G*Power (Faul et al., 2007), using the smallest effect size (*d*_*z*_ = 0.7) in a previous memory updating study (Kong & Fougnie, 2019; Cohen ' s *d*_*z*_ ranging from 0.7 to 1.3). As we were aiming to detect an effect of one of the main modes of attention, we decided that smaller effect sizes would not fulfill that criterion. We estimated that a sample size of 18 participants would yield a power of .80 at alpha level of .05.

All participants reported normal or corrected-to-normal vision. Each participant gave written informed consent before the experiment. The study was approved by the New York University Abu Dhabi Institutional Review Board, and follows the principles laid out in the Belmont Report.

#### Apparatus and stimuli

**Fig. 1:**
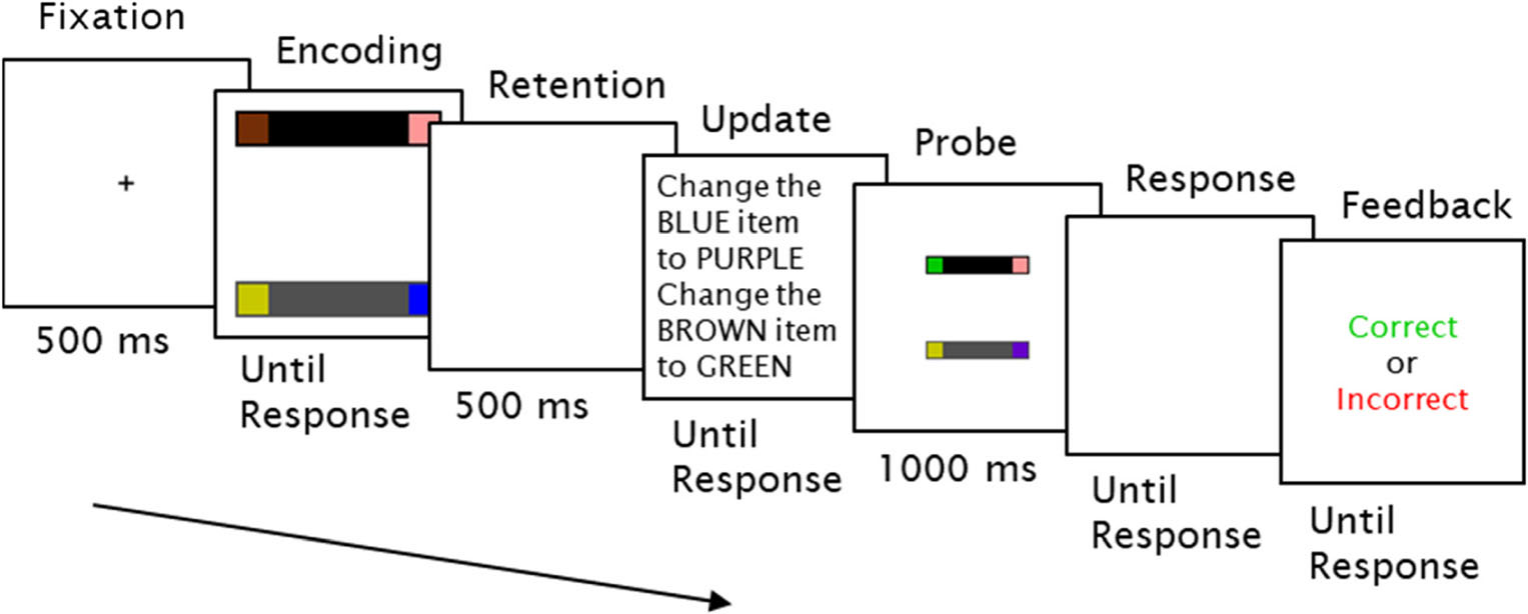
Trial sequence of Experiment 1. Participants first encoded a memory display before updating the colored items in their memory according to instructions. Finally, they responded on whether the probed objects were the same as or different to their updated memory representations. In the probe display, objects were decreased in size by 50% on all trials, and rectangles could appear in the opposite direction or in swapped positions. Crucially, this did not affect the same response, such that participants had to remember the bar properties and not the color positions

The experiment was programmed in MATLAB using the Psychtoolbox extension (Brainard, 1997; Kleiner et al., 2007; Pelli, 1997). All stimuli were displayed against a light grey background on a 24-in. BenQ XL2411 monitor (1,920 × 1,080 pixels) placed 57 cm from the participant. The memory display consisted of two parallel rectangle bars (11.047° × 1.93°), one black and one grey, oriented horizontally or vertically (second frame in Fig. 1). Each end of the bars contained a square (1.61° × 1.61°), the color of which was selected from nine possible options (white, red, green, blue, yellow, purple, pink, orange, brown). The probe display contained two parallel bars (5.52° × 0.97°) with colored squares (0.81° × 0.81°) at each end and in the same orientation as in the memory display.

To exclude the possibility that participants were merely encoding color-location pairings instead of bound objects, we decreased the size of the probe display by 50% and manipulated the position of objects: there was a 50% probability that an object would be spun around, a 50% probability that the other object would be spun around, and a 50% probability that the objects would swap positions with each other such that each colored square had an equal probability of appearing at the four possible locations. Participants were instructed with regard to these potential changes and were told not to base the same or different judgment on these irrelevant changes. On half of the probe displays, the objects would be correctly updated, and on the other half, there was an equal probability that a target square retained its old color, that a target square was changed to a color that was not used during that trial, that diagonal squares swapped positions, or that the bars swapped colors.

#### Design and procedure

A summary of the trial sequence is shown in Fig. 1. Each trial began with the presentation of a fixation cross (length 0.81°, width 0.16°) for 500 ms. To minimize verbal encoding and rehearsal, participants were instructed to repeat the word “COLA” upon the onset of the fixation cross and to continue repeating throughout the trial and to stop only once the memory response was completed. Articulatory suppression was monitored remotely by the experimenter. Afterwards, the memory display was presented and remained on-screen until participants indicated with a mouse click that they had fully memorized the objects. This would ensure that participants had accurate memory of the display. The display was then replaced by a blank screen (500 ms), followed by the updating task. Participants were shown on-screen instructions to update two of the colored squares into two new colors, such as “Change the BLUE item to PURPLE” and “Change the BROWN item to GREEN.” Trials were equally divided between three update conditions: the to-be-selected squares were on the opposite ends of the same rectangle, on adjacent locations of different rectangles, or on diagonal locations of different rectangles. Update times were self-paced.

After participants indicated with a mouse click that they had updated the items mentally, the probe display was presented for 1 s. The presentation time was kept brief to discourage participants from updating during the memory test. Participants responded with a left mouse click if the objects were updated correctly or with a right mouse click if they were updated incorrectly. Participants could start the response upon presentation of the probe display. Upon response, feedback on accuracy was provided by showing “CORRECT” in green or “INCORRECT” in red. The probe display remained on the screen. In the case where the probe was updated incorrectly, we gave additional feedback by outlining the incorrectly updated squares in correct colors or outlining the rectangle bars in red when their colors were swapped.

Participants completed 180 trials divided into seven blocks. The experiment was preceded by 15 practice trials. In addition, to increase motivation, participants received 10 AED bonus if their accuracy rate was above 75% and 20 AED bonus if it was above 85%.

#### Analysis

In order to determine whether it was easier to update the same object, we conducted paired *t*-tests to compare updating performance (probe accuracy and reaction times (RTs) for the self-paced updating period) for equidistant pairs of targets on the same object or on different objects. Since we had no specific hypotheses for the condition involving the diagonal update, it was left out of the analysis. Differences in probe accuracy are assumed to reflect differences in updating (not differences in encoding) since encoding conditions did not differ between conditions. Similarly, longer update RTs are thought to reflect a more difficult updating transition between the first and the second update instruction. Updating times deviating more than 3 standard deviations from the mean were excluded as outliers, leading to a loss of 1.61% of all trials. Below we include analyses with incorrect trials included. However, excluding incorrect trials did not impact the findings.

Our experiment included a manipulation of bar orientation and/or position between the memory and probe displays to discourage spatial encoding. If participants did not rely on a spatial strategy to perform the task, scrambling the locations of objects at test should not disrupt memory performance. To verify this, we analyzed probe accuracy and decision time with 3 (bar rotation: no change, one bar rotated, both bars rotated) × 2 (position swap of bars: no swap, swap) repeated-measures ANOVAs.

### Results and discussion

Mean encoding duration for the initial display was 8.00 s (*SD* = 4.09). We did not further analyze encoding duration as memory displays did not differ across conditions.

#### Updating performance

**Fig. 2:**
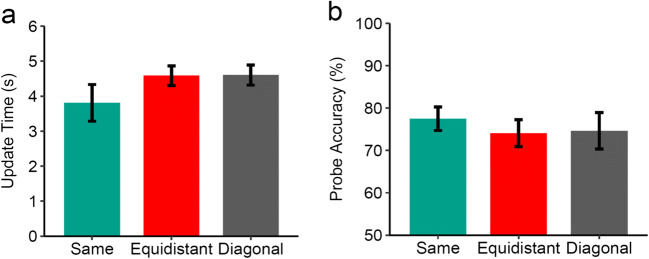
Mean update times and accuracy for Experiment 1 (*n* = 18). Error bars represent 95% within-subjects confidence intervals (O’Brien & Cousineau, 2014)

Mean update times are shown in Fig. 2a. Participants were faster in updating target squares on the same (3.80 s) versus different objects (4.58 s), 95% confidence interval for mean difference, CI [0.13 1.42], *t*(17) = 2.54, *p* = .021, *d*_*z*_ = 0.60. In addition, probe accuracy (Fig. 2b) was higher when targets were on the same (77.50%) versus different objects (74.07%), 95% CI [0.03 6.82], *t*(17) = 2.13, *p* = .048, *d*_*z*_ = 0.50. This rules out the possibility that the observed difference in update time was due to speed-accuracy tradeoff and provides further evidence for a same-object benefit in updating performance. Further, we analyzed the time to make a decision to the probe display to check whether faster updates for the same object were because participants chose to update during the probe display instead of during the instruction display. However, decision times did not differ between same (1.52 s) and different (1.58 s) objects, 95% CI [-0.23 0.10], *t*(17) = 0.80, *p* = .437, *d*_*z*_ = 0.19, suggesting that there were no strategic differences in updating during the probe display.

#### Probe manipulation

We tested whether spatial manipulation of the probe display disrupted performance. Analyses on both probe accuracy and decision time showed no main effects or interactions (all *Fs* < 3.06, *p*s > .098). Thus, presenting objects at a different location or in the opposite direction had little impact on performance, consistent with work showing that memory is relatively unimpaired by irrelevant location changes during the probe display (Logie et al., 2011; Treisman & Zhang, 2006; Udale et al., 2018; Woodman et al., 2012; but see Hollingworth, 2007; Jiang et al., 2000).

To assess whether our probe manipulation was critical to observing the object-based benefits, we analyzed updating performance on trials where the probe display preserved the spatial layout of the memory display (no bar rotation or swaps). There were relatively few trials in this baseline condition (meaning the statistical tests had reduced power). There was no effect in update time, *t*(17) = 1.75, *p* = .098, CI = [-1.61, 0.15], *d*_*z*_ = 0.41, but the trend was toward faster update times for the same (3.81 s) versus different objects (4.53 s). Accuracy did not differ between same (77.0%) and different objects (72.0%), *t*(17) = 0.93, *p* = .365, CI = [-6.37 16.41], *d*_*z*_ = 0.22.

During visual perception, it is faster to shift attention between locations within the same object than across two objects (Egly et al., 1994), thus demonstrating object-based attention. In line with this, we found that updating two features on the same object is faster and more accurate than updating features across two objects despite an equal spatial separation between conditions. This suggests that selection over perceptual and memory representations operate via similar object-based mechanisms.

One potential limitation of Experiment 1 is that the colored squares within each object structure might not have been represented as an object (but see Xu & Chun, 2007, for evidence of object-based processing), but as a “chunk,” such that object-based benefits could instead reflect more efficient updating within a single chunk (Oberauer & Bialkova, 2009). Given how both chunks and objects involve the integration of multiple elements into a unified representation (e.g., Miller, 1956; Thalmann et al., 2019; Wheeler & Treisman, 2002), we are not confident that the two are separate constructs with independent mechanisms. Rather, the difference may reflect the fact that integration of features into objects is less effortful than standard chunking accounts (Luck & Vogel, 1997). Regardless, to provide a stronger test of object-based effects, Experiment 2 used multi-feature objects to better align with most definitions of an object as a binding of different features.

## Experiment 2

Experiment 1 suggested that participants could shift access between equidistant colors on the same versus different objects. Experiment 2 aimed to extend this finding by exploring how attention spreads in a retro-cueing task. On each trial, a pair of Gabors (with color and orientation information) were sequentially presented. A retro-cue then indicated the most relevant object and feature (e.g., color of the first Gabor). Participants reported the color or orientation of a probed item on a continuous response wheel. Of interest is whether attention to a single object feature spreads more towards the uncued feature or the uncued object. The object-based account predicts a benefit for another feature bound to the same object (e.g., Sahan et al., 2020), whereas the feature-based account predicts facilitation for the same feature dimension in another object (Niklaus et al., 2017). Finding a benefit for distinct features within the same object would provide converging evidence for object-based effects in VWM and highlight that such effects are stronger than any putative feature-based mechanisms.

### Method

#### Participants

Eighteen students (12 female, 6 male; mean age 20.5 years, age range: 18–24 years) took part in Experiment 2. Five participants were replaced because mixture modeling analyses (Suchow et al., 2013; Zhang & Luck, 2008) estimated that 50% (or more) of their responses were lapse responses, i.e., made without information about the target.

#### Stimuli

The memory stimuli were Gabor patches (5° diameter, 1° wavelength, spatial frequency of five cycles per stimulus) within a Gaussian envelope (*SD* = 1°). The orientation of the Gabor patches was selected from 180 possible values between 1° and 180° and the color was sampled from a color space consisting of 180 possible values drawn from CIELAB space centered at L* = 54 (luminance), a* = 18, b* = -8, and a radius of 59. The color and orientation of the two memory stimuli in each trial were chosen randomly with the constraint that the values selected for the two stimuli within each trial were at least 30° color steps or 15° orientation steps from each other.

#### Design and procedure

**Fig. 3:**
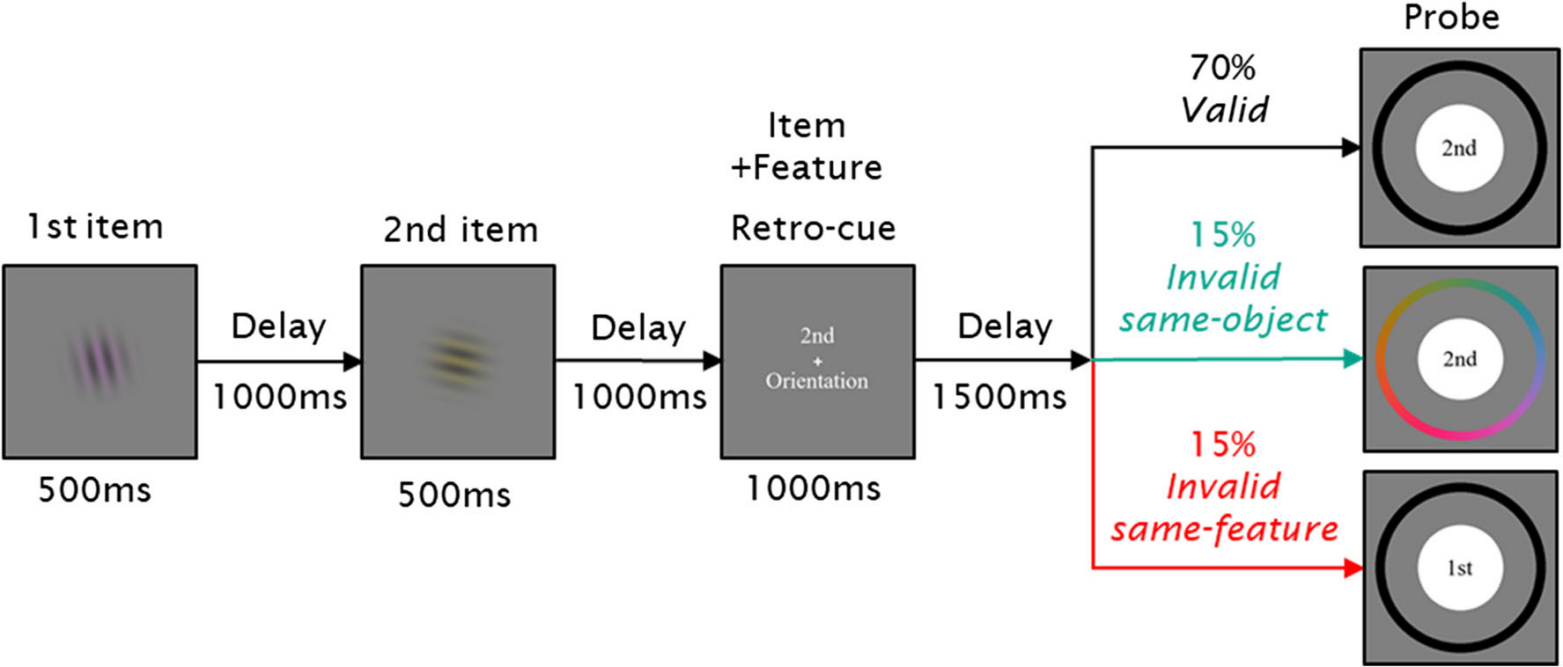
Trial procedure and conditions in Experiment 2. Participants were instructed to memorize both the color and orientation of two Gabor patches that were presented sequentially. During the retention interval, a retro-cue indicated the item (“1st”, “2nd”) and the feature dimension (“Color”, “Orientation”) that would most likely be probed with 70% validity. On invalid trials, the uncued feature of the cued object (*invalid same-object*) and the cued feature of the uncued object (*invalid same-feature*) was probed with equal probability. Participants adjusted the color or orientation of the probe stimulus (color patch or greyscale Gabor grating) to match their memory representation by selecting a feature value on the response wheel

A summary of the trial sequence is shown in Fig. 3. At the start of the trial, a white fixation cross (length 0.5°, width 0.1°) was presented for 200 ms. Then each of the two memory stimuli was serially presented for 500 ms at the screen center, separated by an interstimulus interval of 1,000 ms. Another interval of 1,000 ms followed the second stimulus. The retro-cue was then displayed for 1,000 ms and provided information on both the item (first or second) and the feature dimension (color or orientation) that would be probed with 70% validity. On invalid trials, either the uncued feature of the cued item (*invalid same-object*) or the cued feature of the uncued item (*invalid same-feature*) was tested instead.

After a cue-test delay of 1,500 ms, a white circle appeared (diameter 5°) with the probed item written at the center (i.e., “1st” or “2nd”), followed by a color wheel or a black wheel (radius 5°, width 0.56°) indicating the probed feature. The color wheel was rotated randomly across trials (to prevent participants from remembering the spatial position of the colors), while the same black wheel for orientation was used across trials. Probe instructions in text format (e.g., “1st-Color”) were also provided above the response wheel. When the participant moved the mouse, the white circle was replaced by either a color patch or a greyscale Gabor grating depending on which feature was being probed, in order to provide a visual stimulus to compare with the memory representation. Participants were instructed to adjust the patch’s color or orientation as precisely as possible by selecting a value on the wheel. The response was unspeeded. The patch was updated according to the angular position of the mouse cursor, and a black line outside the response wheel indicated the currently selected value. Participants clicked on the mouse to provide the response, after which feedback was presented for 1.5 s. In the feedback display, a color patch or a greyscale grating at the center showed the true feature value for the probed item, while a red line and a black line marked, respectively, the correct position and the participant’s response on the response wheel. Feedback in degrees from the target was also provided. The following trial began after an intertrial interval of 1 s.

Participants completed 400 trials separated into ten blocks of 40 trials each. The experiment consisted of 280 *valid* trials (2 item cue (first, second) × 2 feature cue (color, orientation) × 70 repetitions) and 60 trials each for *invalid same-object* and *invalid same-feature* conditions (2 item cue × 2 feature cue × 15 repetitions). Prior to the study, participants completed ten practice trials.

#### Analysis

We computed response error by calculating the angular distance between the true color or orientation for the probed item and the reported feature value. To assess memory performance, we performed a 3 (cue condition: *valid*, *invalid same-object*, *invalid same-feature*) × 2 (probe item: first, second) × 2 (probe feature: color, orientation) repeated-measures ANOVA on the mean absolute response error and followed up significant effects or interactions with contrasts.

To examine possible sources of memory error, we analyzed the response error with the standard mixture model (Suchow et al., 2013; Zhang & Luck, 2008) using maximum-likelihood estimation. This model provides estimates for two parameters: lapse rate and standard deviation (*SD*). Lapse rate represents the proportion of responses made without information about the probed feature or due to random guesses. The *SD* parameter reflects the precision of items stored in memory (higher *SD* equals worse precision). Because our analyses of mean response error only showed significant effects of cue condition and probe feature, we collapsed across probe item conditions and fitted the data separately for each participant and for each cue and probe feature combination. We then performed repeated-measures ANOVAs, with factors cue condition and probe feature, on lapse rate and the *SD* parameter.

### Results and discussion

**Fig. 4:**
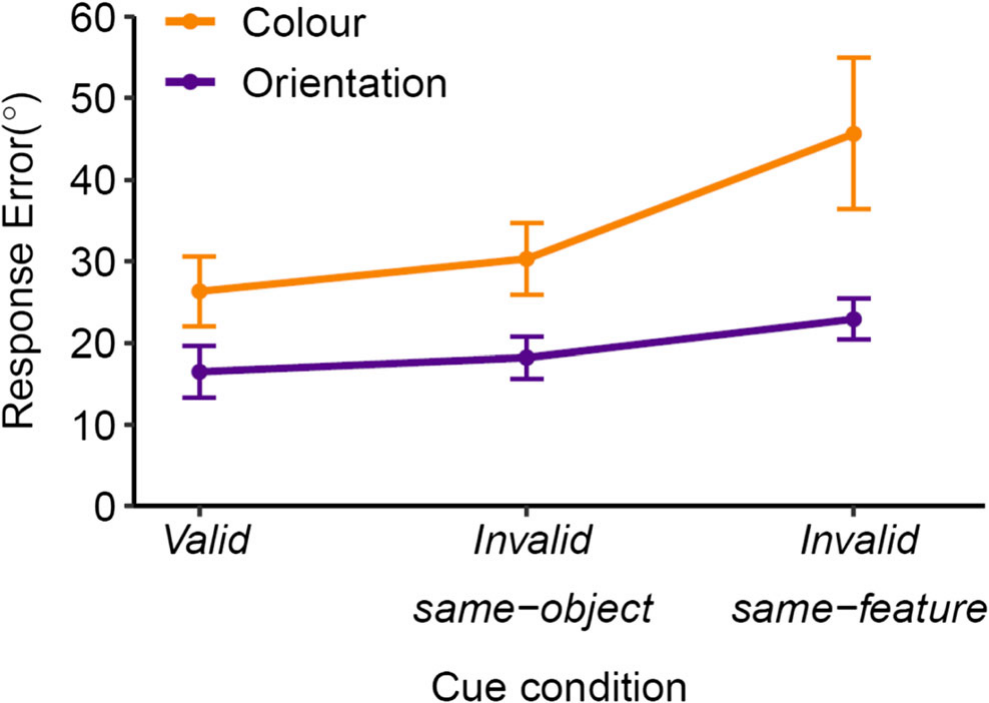
Mean absolute error in Experiment 2 (*n* = 18). Error bars represent 95% within-subjects confidence intervals (O’Brien & Cousineau, 2014)

Analyses on mean absolute response error (Fig. 4) revealed a main effect of cue, *F*(2,34) = 11.94, *p* < .001, η_p_^2^ = .413. Memory error was higher for color (34.10) versus orientation (19.19), *F*(1,17) = 67.51, *p* < .001, η_p_^2^ = .799. There was no effect of probe item, *F*(1,17) = 1.35, *p* = .262, η_p_^2^ = .073, suggesting that serial position did not influence performance. There was an interaction effect between cue and probe feature, *F*(2,34) = 6.69, *p* = .004, η_p_^2^ = .282. All other interactions were not significant (all *Fs* < 1.34, *p*s > .274).

To understand the main effect of cue, we used three Bonferroni-adjusted planned contrasts, averaged across probe feature and probe item. Response errors were smaller on *valid* trials (21.40) than on both the *invalid same-feature* (34.28), *t*(17) = 3.71, *p* = .005, *d*_*z*_ = 0.88, and *invalid same-object* trials (24.25), *t*(17) = 2.92, *p* = .029, *d*_*z*_ = 0.69, demonstrating that cues were effective. Most relevant to our hypothesis, response errors were smaller in the *invalid same-object* versus *invalid same-feature* condition, *t*(17) = 3.17, *p* = .017, *d*_*z*_ = 0.75, demonstrating an object-based benefit. To understand the interaction between cue and probe feature, two orthogonal simple contrasts compared the two invalid conditions separately for each probe feature. Response errors were smaller in the *invalid same-object* versus *invalid same-feature* condition for both color (*M*s = 30.30 and 45.67), *t*(17) = 2.90, *p* = .010, *d*_*z*_ = 0.68, and orientation (*M*s = 18.19 and 22.89), *t*(17) = 3.24, *p* = .005, *d*_*z*_ = 0.76, suggesting that the object-based benefit was not driven by effects for one feature.

#### Mixture modelling

Report errors were turned into measures of lapse rate and imprecision (*SD*) using mixture modelling (Zhang & Luck, 2008). For lapse rate, we found a main effect of cue*, F*(2,34) = 13.25, *p* < .001, η_p_^2^ = .438. Bonferroni comparisons revealed that *valid* cues (11.94%) reduced lapse rates relative to *invalid same-feature* cues (34.87%), *t*(17) = 4.08, *p* = .002, *d*_*z*_ = 0.96, but did not reduce lapse rates relative to *invalid same-object* cues (16.33%), *t*(17) = 1.95, *p* = .203, *d*_*z*_ = 0.46. More importantly, we found an object-based benefit: *invalid same-object* cues reduced lapse rates relative to *invalid same-feature* cues, *t*(17) = 3.36, *p* = .011, *d*_*z*_ = 0.79. There was no effect of probe feature, *F*(1,17) = 0.77, *p* = .393, η_p_^2^ = .043, and there was no interaction, *F*(2,34) = 0.01, *p* = .987, η_p_^2^ = .001.

In contrast, the *SD* parameter did not differ across cue conditions (*M*s = 27.83, 28.45, and 26.23), *F*(2,34) = 0.55, *p* = .581, η_p_^2^ = .031. There was better precision for color (23.32) than orientation (31.69), *F*(1,17) = 20.92, *p* < .001, η_p_^2^ = .552. There was no interaction, *F*(2,34) = 1.68, *p* = .202, η_p_^2^ = .090. These results might suggest that the object-based benefit was due to lower probability of random responses rather than enhanced memory precision. However, we would like to caution against overinterpreting the mixture modelling results. There is a limited number of trials for the two invalid conditions, which can make the modelling results less reliable. Furthermore, recent papers have questioned whether the lapse rate is representative of a lack of information when responding (Schurgin et al., 2020; Taylor & Bays, 2020).

We found better memory for an uncued feature in the same object than for the same feature dimension in the uncued object. This suggests that object-based attention can be distinguished from, and may even be stronger than, feature-based attention in VWM. However, this does not necessarily argue against the existence of feature-based selection. Rather our finding that valid cues improve memory recall relative to invalid cues implies that internal prioritization operates at both the object and the feature level (Niklaus et al., 2017; Park et al., 2017; Ye et al., 2016).

## General discussion

Attentional mechanisms allow us to focus on relevant information in a complex visual environment. Attention is not a monolithic process, but includes space-based, feature-based, and object-based mechanisms. Further, growing evidence suggests that attention also influences information stored in VWM. The current study investigated whether object-based attentional effects also arise in VWM. We found that selecting part of an object extends an attentional benefit to another location or feature of the same object. These results parallel findings in object-based attention and suggest that object-based representations can guide selection in VWM, as they do in perception.

Our findings add to existing studies showing similar object-based selection mechanisms in perception and VWM (Awh et al., 2001; Bao et al., 2007; Gao et al., 2017; Hajonides et al., 2020; Matsukura & Vecera, 2009; Peters et al., 2015; Sahan et al., 2020; Woodman & Vecera, 2011). For example, Peters et al. (2015) found that participants were faster when shifting between locations on a screen when those locations belonged to a previously displayed object than when they belonged to two objects. Experiment 1 extended these results by showing that object-based effects can occur even when the attentional process is entirely internal. Experiment 2 further showed that selection of an object feature also spreads to the entire object, perhaps more so than the same feature will spread to other objects, consistent with recent neural evidence (Sahan et al., 2020). Crucially this finding reflects differences in how object and feature representations are prioritized in VWM. This explains why object retro-cues have a larger benefit compared to feature retro-cues (Hajonides et al., 2020).

Nonetheless, it should be noted that the current study explicitly encouraged object-based encoding. We reasoned that object-based selection in VWM requires storage of information in an object-based format. Although work has proposed that there is automatic encoding and storage of objects in VWM (e.g., Luck & Vogel, 1997; Shen et al., 2013; Vogel et al., 2001), recent studies suggest that object-based encoding occurs primarily when the task encourages this type of encoding (Hardman & Cowan, 2015; Qian et al., 2019; Vergauwe & Cowan, 2015; but see Ecker et al., 2013; Gao et al., 2016; Shin & Ma, 2016; Swan et al., 2016). Therefore, to maximize the likelihood of finding object-based selection effects, our task was designed to encourage the use of object-based representations. Specifically, we scrambled the location of objects in Experiment 1 and placed objects at the same location in Experiment 2. Considering how strongly we encouraged participants to hold integrated object representations instead of feature-location bindings, we do not claim that object-based selection is mandatory, and such effects might be contingent on the existence of object-based representations. In fact, differences in encoding strategies could possibly explain why other studies have failed to observe object-based selection in VWM (Ko & Seiffert, 2009). Although we discouraged spatial encoding to rule out space-based explanations of object-based benefits, we do not imply that spatial relationships between items are not important. Indeed, studies have shown interference from spatially close items in memory (Bays, 2016; Tamber-Rosenau et al., 2015), which together with the present findings suggest that VWM encodes both object and spatial representations of the environment (e.g., Golomb et al., 2014; but see Allen et al., 2015; Dowd & Golomb, 2019).

Here we adapted paradigms of object-based attention to show that similar mechanisms also operate in VWM. Selecting one aspect of an object facilitates selection of the entire object, even after controlling for spatial and feature-based attention. Our results show another way in which perceptual and memory representations are selected similarly, further supporting the view that there is considerable overlap between working memory and attention. The present work suggests that we can learn much about the properties of selection and updating in VWM from exploring the degree to which influential paradigms in visual attention apply similarly to representations held in the mind.
